# Understanding the Impact of Dietary Cholesterol on Chronic Metabolic Diseases through Studies in Rodent Models

**DOI:** 10.3390/nu10070939

**Published:** 2018-07-21

**Authors:** Ángela Vinué, Andrea Herrero-Cervera, Herminia González-Navarro

**Affiliations:** 1 INCLIVA Health Research Institute, 46010 Valencia, Spain; m.angela.vinue@uv.es (Á.V.); anhecer@alumni.uv.es (A.H.-C.); 2 CIBER de Diabetes y Enfermedades Metabólicas asociadas (CIBERDEM), 28029 Madrid, Spain

**Keywords:** dietary cholesterol, fatty liver disease, animal models, atherosclerosis, inflammation

## Abstract

The development of certain chronic metabolic diseases has been attributed to elevated levels of dietary cholesterol. However, decades of research in animal models and humans have demonstrated a high complexity with respect to the impact of dietary cholesterol on the progression of these diseases. Thus, recent investigations in non-alcoholic fatty liver disease (NAFLD) point to dietary cholesterol as a key factor for the activation of inflammatory pathways underlying the transition from NAFLD to non-alcoholic steatohepatitis (NASH) and to hepatic carcinoma. Dietary cholesterol was initially thought to be the key factor for cardiovascular disease development, but its impact on the disease depends partly on the capacity to modulate plasmatic circulating low-density lipoprotein (LDL) cholesterol levels. These studies evidence a complex relationship between these chronic metabolic diseases and dietary cholesterol, which, in certain conditions, might promote metabolic complications. In this review, we summarize rodent studies that evaluate the impact of dietary cholesterol on these two prevalent chronic diseases and their relevance to human pathology.

## 1. Introduction

The role of dietary cholesterol has been studied extensively in humans and experimental animals. Studies in animal models have classically pointed to dietary cholesterol as responsible for several chronic diseases, including cardiovascular disease (CVD) and non-alcoholic fatty liver disease (NAFLD). However, recent clinical and epidemiological studies have changed perspectives on dietary cholesterol [1].

In humans, nearly 70% of overweight subjects and up to 90% of obese people will develop NAFLD [2], leading to the accepted view that the main risk factor for NAFLD development is overweight status or obesity. NAFLD, also referred to as fatty liver or hepatic steatosis, is asymptomatic per se, but progression toward hepatic fibrosis and inflammation induces non-alcoholic steatohepatitis (NASH), a dysfunctional hepatic state. In some cases, NASH can develop into hepatocarcinoma (HCC). However, NASH was initially described as developing via the “two-hit” model hypothesis, which included a first step of hepatic fat accumulation, followed by liver injury through activation of hepatic stellate cells, by oxidative stress or activation of cytokine-signaling pathways [3]. Currently, the hypothesis has evolved toward a “multiple-hit” hypothesis with many factors involved in the progression from simple hepatic steatosis to NASH [4]. Defective insulin signaling (such as in insulin resistance (IR) and type 2 diabetes (T2DM)), as well as genetic and environmental factors are some of the determinants in the transition from an asymptomatic status toward severe disease. Clinical data from NAFLD subjects with and without obesity led to the association between dietary cholesterol and progression of the disease [5,6,7]. Of note, studies in mouse models treated with the so-called “atherogenic diet” have shown that dietary cholesterol could be one of the main factors inducing the hepatic injury that leads to NASH [8]. Interestingly, increased cardiovascular risk in subjects with chronic NAFLD [9], especially in co-occurrence with diabetes, has also been described [10].

Atherosclerosis, the main cause of CVD, is considered a chronic inflammatory disease resulting from the interaction between cholesterol-containing particles (mainly low-density lipoproteins, LDLs) and elements of the arterial wall and immune cells. The process leads to initial fatty streak lesions consisting of lipid-loaded macrophages or foam cells in the subendothelial space [9]. Macrophages, which initially have a pro-resolving M2 phenotype, keep lesions small, but perpetuation of this process soon switches macrophages toward a proinflammatory M1 phenotype. T cells also recruited into the lesions play a key role modulating the inflammatory process by secreting pro and anti-inflammatory stimuli [10]. A deranged balance between proinflammatory T helper (Th1) and anti-atherogenic regulatory T (Treg) cells promotes a chronic unresolved inflammatory process that accelerates cell death events and plaque rupture, leading to thrombotic and ischemic events [11].

Because atherosclerosis is initiated by lipid particles, high circulating cholesterol levels, mostly LDL-cholesterol (C) particles, constitute the main risk factor for developing atherosclerosis. Reducing LDL-C is a primary goal in atherosclerosis prevention [12] and it was long believed that restricting dietary cholesterol intake would significantly reduce disease incidence. However, plasma cholesterol levels ultimately depend on the activation status of pathways that modulate hepatic function such as bile acid synthesis, cholesterol secretion into bile, and cholesterol pool storage [13]. In fact, many clinical and epidemiologic studies have shown inconsistency among results. In some studies the effect of dietary cholesterol on serum total circulating cholesterol was small, while in others the LDL:HDL ratio, an index for atherogenic risk assessment, remained unchanged [14]. In yet other studies, a discrete shift in the size of LDL particles was found, with no change to CVD risk [15]. Moreover, although dietary cholesterol might in some conditions aggravate human CVD, other dietary components such as the presence of saturated fats, lifestyle issues, genetics, and environmental factors (i.e., pollution) also modulate the effect of dietary cholesterol in LDL-C levels and therefore on vascular disease [13,15]. Indeed, a whole body of evidence from studies in humans has shown that it is dietary saturated fat which best correlates with circulating serum LDL-C levels and with incidence of CVD [12]. Thus, dietary studies limiting saturated fat intake showed efficiency in reducing LDL-C in plasma [12]. On the other hand, given that foods enriched in saturated fat also contain high amounts of cholesterol, it has been difficult to understand the specific contribution of dietary cholesterol.

CVD and NAFLD share hepatic homeostasis derangement, which might be driven by differential expression of key metabolic genes. The liver X receptors (LXRs) are nuclear receptors activated by oxysterols which are derivatives of cholesterol, therefore acting as cholesterol sensors [16,17]. Upon activation of LXRs, a number of main cholesterol metabolic genes are transcribed, which mainly modulate the cholesterol pool in the liver and avoid cholesterol overload in peripheral tissues. LXRs in macrophages enhance the expression of ABCA1, promoting reverse cholesterol transport by HDL. In the liver, LXR promotes cholesterol secretion into the bile by activating the expression of CYP7A1, a key enzyme in bile acid biosynthesis, and adenosine triphosphate-binding cassette transporters G5 and G8 (ABCG5 and ABCG8, respectively), which mediate efflux of hepatic cholesterol into the bile. Importantly, activation of ABCG5/G8 transporters in the intestine also facilitates fecal cholesterol excretion and diminishes dietary cholesterol absorption [17]. LXRs are also involved in plasma cholesterol uptake by the liver through modulation of low-density lipoprotein receptor (LDLr) expression by Idol, which targets LDLr for degradation. Given that LXRs modulate many steps of cholesterol homeostasis, alteration of their functionality affects endogenous cholesterol disposal and pools, as well as dietary cholesterol absorption. Of note, biliary acids such as cholic acid, which has been used in dietary cholesterol studies, might aggravate the effects of dietary cholesterol. By binding to the farnesoid X receptor (FXR), cholic acid increases the absorption of dietary cholesterol from the intestine and might repress CYP7A1, which facilitates cholesterol accumulation in the liver [18]. Alteration of these pathways has been observed in the progression of both CVD and NAFLD [16,17].

In this review, we summarize available evidence from rodent models describing the relevance of dietary cholesterol in the two abovementioned related diseases. There are a high number of studies in rodent models that routinely use cholesterol-containing diets. However, to include the studies in the present review, the selection criterion was to specifically address the contribution of dietary cholesterol using different concentrations and/or combinations with fat, with a comparison to diets without cholesterol. Of note, in these studies, dietary cholesterol corresponds roughly to 20,000 mg/day, which is 60-fold the amount of cholesterol consumed daily by humans. Therefore, the findings in these studies may not directly always translate into human pathology process.

## 2. Dietary Cholesterol in Non-Alcoholic Fatty Liver Disease in Rodent Models

The understanding of the mechanisms behind NAFLD/NASH progression has been stablished in studies examining animal models fed with different dietary combinations of fat, cholesterol, and cholate. In the following sections, we summarize rodent studies describing the impact of dietary cholesterol as one of the key determinants in NAFLD progression. These models are summarized in Table 1 and Table 2.

### 2.1. Studies in Rodent Models Using Different Cholesterol-Rich Diets

Described in this section are studies using different cholesterol and fat contents in rats and wild-type mice to analyze their impact in NAFLD.

#### 2.1.1. Studies in Rats

Sprague–Dawley rats fed an atherogenic diet containing 40% fat and 1.25% cholesterol promoted hepatic accumulation of triglycerides and cholesterol compared with regular diet-fed rats [19]. An atherogenic diet was also associated with a decrease in the liver of the farnesoid X receptor (FXR), 3-hydroxy-3-methylglutaryl coenzyme A reductase (HMG-CoA-r), squalene synthase, and ABCG8, suggesting that dietary cholesterol could promote fatty liver disease by modulating expression of these genes. Also in Sprague–Dawley rats, a comparison of different diets containing fat alone or in combination with 1.25% or 2.5% cholesterol indicated that dietary cholesterol plays a role in the inflammatory process of hepatic steatosis and diminishes hepatic metabolic gene expression of microsomal triglyceride transfer proteins, carnitine palmitoyl transferase activity, and ABCG5 [20].

In a recently described stroke-prone, spontaneously hypersensitive SHRSP5/Dmcr rat model, the pro-inflammatory TNFα cytokine and p50/p65 nuclear factor kappa B (NFkB) pathway mediated inflammatory signals and oxidative stress characteristic of the hepatic fibrotic and inflammatory status of NASH [21] when fed a diet rich in cholesterol. Moreover, authors identified serum K18Asp396 levels, a neoepitope generated during cleavage of keratin 18 by caspases, as a potential biomarker of hepatic necrosis [22]. In another study, examination of SHRSP5/Dmcr rats fed a high-cholesterol diet showed steatosis as well as inflammatory and fibrotic status in a time-dependent manner, characterized by eosinophilic inclusion bodies and mega-mitochondria, which are signs of advanced NASH [23].

#### 2.1.2. Studies in Mice

In wild-type (WT) C57Bl/6J mice, atherogenic diets containing 1.25% cholesterol and two different amounts of fat (7.5% and 60%) induced oxidative stress, fibrosis, steatohepatitis, and cellular ballooning, which is an important histological feature in human NASH, as compared with mice fed a control diet [8].

Savard and colleagues reported that diets containing either 15% of fat (high fat, HF) or 1% cholesterol (high-cholesterol, HC) produced hepatic fat accumulation without signs of inflammation, liver damage, or fibrosis [24]. However, dietary combination of 15% of fat and 1% cholesterol resulted in severe steatosis, inflammation, and fibrosis resembling that of human NASH, indicating that fat and cholesterol synergize to promote steatohepatitis [25].

DNA microarray technology was employed in C57BL/6J mice to compare the effect of different diets containing fat, cholate and/or cholesterol, or these nutrients in combination. The study revealed specific genes up- and down-regulated by cholesterol [26]. Interestingly, in this study mice developed hepatic steatohepatitis when fed cholesterol only, but hepatic stellate cell activation, a key event in the progression toward NASH, was only observed when cholic acid and cholesterol were combined in the diet. Among the activated genes were serum amyloid A (SAA) family genes, histocompatibility antigens, interleukin-2 receptor γ (*Il2rg*), small inducible cytokine B9 (*Scyb9*), and SAM domain and HD domain 1 (*Samhd1*). Interestingly, among downregulated genes were some that used the hepatic cholesterol pool, such as aquaporin-8, involved in bile secretion, *Cyp17a1*, a key enzyme in steroid biosynthesis, and apolipoprotein A-IV, a component of high-density lipoproteins. Thus, dietary cholesterol induces gene expression changes in the liver that facilitate cholesterol accumulation.

Other studies with long dietary treatments or using dietary combinations with cholate led to mouse models with hepatic tumors and identified key inflammatory genes. Treatment of mice with an atherogenic diet containing 1.25% cholesterol, 0.5% cholic acid, and 16% fat for 3 weeks induced mononuclear leukocyte infiltration in the liver, hepatic steatosis, and increased expression of inflammatory genes such as *MCP1*, *RANTES*, and *MIP2*. Of note, toll-like receptor (TLR)4-deficient mice displayed 30% attenuation in hepatic injury enzymes, 50% reduction in leukocyte infiltration, and a fourfold reduction in chemokine expression [27]. In another study, long-term treatment of mice with a diet composed of 15% milk fat, 1.5% cholesterol, and 0.1% cholic acid for 55 weeks resulted in hypercholesterolemia, hepatic steatosis, fibrosis, and tumor formation in the form of focal nodular hyperplasia. These were associated with elevated plasma monocyte chemotactic protein 1 (MCP1) levels and hepatic expression of platelet-derived growth factor b (PDGF-B) protein; thus these proteins influence the severity of disease [28]. Similarly, mice fed a high-fat, high-cholesterol diet with a high sugar supplement showed hepatic steatosis at the early stage of 4 weeks of treatment. This developed into fibrosis and characteristics of NASH at week 27 with enhanced levels of MCP1, TNFα, and IL-1β pro-inflammatory cytokines [29]. Tumor development occurred in 41% of mice, with increased p53 expression and a change in the macrophage polarization toward an M1 macrophage pro-inflammatory phenotype.

Interestingly, genetic inactivation of LXRα/β in mice increased the number of proinflammatory F4/80+CD11b+ macrophages in the liver, suggesting that deranged cholesterol homeostasis that affects LXR might directly activate inflammatory pathways and immune responses [43].

By using a sophisticated metabolomic approach, Tu and co-workers have recently shown that dietary cholesterol promotes NAFLD and NASH even in the absence of obesity or increase in abdominal fat [30]. Mice fed a high-fat high-cholesterol and cholate diet for three weeks displayed hepatic pathology similar to NAFLD and NASH. The lipidomic and metabolic profiling in the liver led to the conclusion that the mechanisms leading to disease were driven by elevated free cholesterol, cholesterol esters and cholic acid, as well as changes to metabolism of sphingomyelins and phosphatidylcholines.

### 2.2. Studies in Mice Deficient in Lipid Metabolism Genes in Combination with Cholesterol-Rich Diets

Studies with genetically modified mice deficient in lipid metabolism genes fed with diets containing different amounts of cholesterol have also shed light in the role of dietary cholesterol in NAFLD progression. In this section, investigations with mice deficient in lipases (ATGL and HL), LDLr, and apolipoprotein E (apoE), as well as Alms1 mutant (foz/foz) and diabetes NOD.B10 mouse models are summarized.

#### 2.2.1. Studies in Lipase-Deficient Mice in Combination with Cholesterol-Rich Diets

Lipase deficiencies usually result in hypertriglyceridemia, which under certain conditions may promote hepatic lipid accumulation and steatosis. Thus, hepatic depletion of adipose triglyceride lipase (ATGL) in mice led to severe hepatic steatosis in several studies [44,31]. One of the mechanisms by which ATGL-deficiency diminished hepatic homeostasis was activation of inflammatory pathways [32] probably by a peroxisome proliferator-activated receptor α (PPARα)-mediated mechanism [33]. Consistent with these studies, hepatic ATGL overproduction decreased hepatic steatosis and improved liver insulin sensitivity [34].

Similarly, hepatic lipase (HL) deficiency, in combination with a high-fat high-cholesterol diet, also led to dyslipidemia which included hypertriglyceridemia, increased NEFA and hypercholesterolemia. These changes were accompanied by hepatic steatosis and liver inflammation shown as enhanced content in F4/80+ macrophages and increased MCP1 hepatic expression [35]. Significantly, these changes were also accompanied by augmented circulating levels of MCP1 and the Th17 T-cell subset. These findings are in line with previous research showing that pharmacological suppression of Th17 cell differentiation or genetic ablation of the IL-17A receptor in myeloid cells prevented NASH and hepatocarcinoma [45,46]. In this same study, when fed a regular chow diet, age-matched HL-deficient mice did not develop fatty liver or hepatic inflammation, pointing to dietary intervention with a fat content of 10.8% fat and 0.75% cholesterol as the cause of NAFLD [35]. In addition, activation of c-Jun N-terminal kinase (JNK) in liver was observed, which is one of the main drivers in NASH development [47,48]. Surprisingly, a previous study reported decreased BW gain and reduced hepatic steatosis in HL-deficient mice when fed an obesogenic diet containing 21% of fat and 0.15% of cholesterol [36]. The seeming discrepancy between these two studies, might be related to the different impact of the dietary components in the circulating lipid levels. Thus, in the study by Chiu et al. [36] dietary regimen did not change cholesterol or FFA levels while in the study by Andrés-Blasco and colleagues the diet, with less fat but higher levels of dietary cholesterol, produced an important impact on circulating lipid levels and therefore on hepatic fat accumulation.

Comparison of these two later studies therefore points toward the capacity of dietary cholesterol to modulate lipid levels and inflammation as a determinant in NASH development, rather than dietary lipid content alone.

#### 2.2.2. Studies in Genetically Modified Mice in Combination with Cholesterol-Rich Diets

Studies performed in mouse models of hyperlipidemia have also contributed to the understanding of dietary cholesterol in diet-induced NASH.

##### Studies in LDLr-Deficient Mice

Hyperlipidemic low-density lipoprotein receptor (LDLr)-deficient and apoE2 knock-in mice display hepatic steatosis with inflammation when fed a high-fat diet with cholesterol compared with WT mice [3]. However, omission of cholesterol in the diet in these genetically-modified mice produced fatty liver but prevented inflammation. Foamy and bloated Kupffer cells were also reported in cholesterol-fed mice, while absence of cholesterol in the high-fat diet prevented hepatic foam cells, suggesting that cholesterol overload might induce stress in hepatic cells. These results are consistent with research describing that free cholesterol deposition within hepatocytes induces hepatocyte apoptosis and necrosis through activation JNK1 inflammatory pathway [49].

In another study, LDLr-deficient mice fed with a diet enriched in fat, carbohydrate, and cholesterol developed NASH characterized by macrovesicular steatosis, inflammatory cell foci, and fibrosis, while omitting cholesterol from the same diet prevented these characteristics [37]. Moreover, comparison between these two treatments showed that dietary cholesterol also promotes hepatic macrophage infiltration, apoptosis, and oxidative stress. This is consistent with the hypothesis that upon lipotoxic stimulus, such as high dietary cholesterol, Kupffer cells secrete cytokines and reactive oxygen species (ROS), inducing inflammation and oxidative stress [38].

##### Studies in NOD.B10 Mice

Similarly, in Alms1 mutant (foz/foz) and wild-type diabetes NOD.B10 mouse models of obesity, adding dietary cholesterol produced hepatic free cholesterol accumulation accompanied by increased macrophages, liver apoptosis, and fibrosis. These were prevented by removal of cholesterol from the diet [39].

##### Studies in apoE-Deficient Mice

In apolipoprotein E (apoE)-deficient mice, treatment with a Western-type diet containing 1.25% of cholesterol for 7 weeks led to a phenotype resembling that of human NASH. These included hepatic fibrosis, upregulation of cytokines such as transforming growth factor β (TGFβ), increased formation of hepatic collagen and activation of hepatic stellate cells [40].

Interestingly, a study showed a differential effect of Western-type diet (20% of fat and 0.25% of cholesterol) on hepatic steatosis in apoE-/- and LDLr-/- mice. Thus, while LDLr-/- mice developed hepatic steatosis and obesity, apoE-/- mice displayed enhanced macrophage content and inflammatory nodules, indicating differential response of the two genetically modified mice to high-fat high-cholesterol diets [41].

Double deficiency in apoE and LDLr has been used to study steatohepatitis and associated tumorigenesis. To this end in the study, apoE-/-LDLr-/- and WT mice were kept for 35 weeks on a Western diet containing 5% cholesterol and 21% or regular chow control diet [42]. Comparison among the groups demonstrated that double-deficient mice developed increased hepatic steatosis, macrophage and T cell infiltration, fibrosis and hepatocellular injury. Some animals also developed liver damage in the form of tumorigenesis. These changes were accompanied by hepatic ROS accumulation, JNK activation, and induction of peroxisome proliferator-activated receptor (PPAR)-*α* which is of relevance as previous studies have pointed to this as determinant in hepatic steatosis and inflammation.

## 3. The Role of Dietary Cholesterol in the Development of Atherosclerosis in Animal Models

The first assumption of dietary cholesterol contribution to CVD had its foundation in animal models which, unlike humans, display a dramatic increase in serum cholesterol levels when fed a high-cholesterol content diet [50]. Human atherosclerosis develops with moderate increases of LDL-C circulating levels, but in animal models, and especially in mice, atherogenic hypercholesterolemia has been achieved by using extreme amounts of cholesterol in the diet and/or in combination with cholic acid. Although this approach might not translate to human pathology cases, feeding animal models with cholesterol-enriched diets has been highly valuable for obtaining mechanistic insight into the molecular processes of the disease. Moreover, genetically-modified mice have been the most commonly used models to study cholesterol metabolism and atherosclerosis. The main studies directly relating dietary cholesterol and atherosclerosis are described in the next sections and are summarized in Table 3.

### 3.1. Impact of Dietary Cholesterol on Atherosclerosis in Mouse Models with an Historic Perspective

Mice are highly resistant to developing atherosclerosis mainly due to the absence of proatherogenic lipoproteins. In fact, adding fat and cholesterol is not sufficient to induce atherosclerosis, which can be only achieved by adding cholic acid to the diet in combination with high amounts of fat and cholesterol. Thus, in 1968 the Wissler laboratory reported the impact of dietary cholesterol on mouse atherosclerosis by using a diet containing 30% fat, 5% cholesterol, and 2% cholic acid. Feeding mice with the diet increased serum cholesterol levels and induced atheroma lesions but was highly toxic and produced extreme hepatic damage [51].

In 1987, a study by the Beverly Paigen laboratory introduced a less toxic atherogenic diet by combining 1.25% cholesterol, 15% fat, and 0.5% cholic acid. Mice fed with this diet for 14 weeks displayed a reduction in anti-atherogenic HDL-C, with discrete hypercholesterolemia and atheroma lesions in the vasculature in the C57BL/6 mouse strain [52]. These dietary studies underscored the resistance of mice to developing vascular pathology as only one of the three mouse strains tested, which included C57BL/6, BALB/c, and C3H, developed vascular lesions. Moreover, a comparison of these strains led to the identification of a number of genetic loci predisposing to atherosclerosis development such as Ath1, located in chromosome 1 [76].

To evaluate the specific contributions of the atherogenic diet components in WT C57BL mice, Vergnes and colleagues performed a DNA microarray study in the liver of mice treated with different diets. The diets used were a chow diet, a typical atherogenic diet containing 1.25% cholesterol, 0.5% sodium cholate, and 7.5% cocoa butter, and three other diets which omitted one of the three components of the mentioned atherogenic diet [26]. Gene expression analysis demonstrated that dietary cholesterol induces the expression of inflammatory genes, while cholate promotes the expression of extracellular matrix deposition genes such as collagen and the activation of stellate cells.

The resistance observed in mice to developing atherosclerosis rely on several metabolic differential characteristics. Thus, circulating levels of LDL in mice are not detectable due to the high LDL-clearance rate by the liver. Thus, most of the cholesterol is carried by HDL particles [77]. Another difference is the absence in mice of cholesterol-ester transport protein (CETP), which transfers cholesteryl esters from HDL to LDL and VLDL in exchange for triglycerides [78]. Given these differences between mice and humans, most of the cholesterol metabolism and atherosclerosis studies are currently performed in genetically-modified mice. These mouse models are mice deficient in key metabolic proteins such as LDLr [53] and apoE [79,63] or transgenic mice overexpressing atherogenic proteins such as the isoform ApoE3 Leiden, a apoE isoform with low affinity to hepatic receptors [80] or apoB100, an apolipoprotein present in all proatherogenic particles.

### 3.2. Impact of Dietary Cholesterol in Genetically-Modified Mouse Models of Atherosclerosis

Current knowledge of the mechanisms and risk factors for atherosclerosis development have been obtained from research on genetically-modified mice using different approaches including combination with different diets to induce hypercholesterolemia [81]. Some studies used dietary cholesterol to accelerate atherosclerosis formation thus linking dietary cholesterol and plaque development. Moreover, the combination of several genetic modifications has led to the identification of up to 827 mouse atherosclerotic genes. Some of these findings have been translated into human pathology and helped to develop therapeutic targets which have received excellent review elsewhere [81]. Below are shown studies using different dietary cholesterol contents in genetically-modified mouse models.

#### 3.2.1. Studies in LDLr-Deficient Mice

Genetic inactivation of LDLr in mice delays LDL clearance and increases LDL-C levels in plasma up to 300 mg/dL [53]. This gene defect is analogous to familial hypercholesterolemia in humans. In the absence of dietary cholesterol, LDLr-deficient mice did not display atherosclerosis but the combination with a diet enriched with cholesterol containing 1.25% cholesterol, 7.5% cocoa butter, 7.5% casein, and 0.5% cholic acid resulted in severe hypercholesterolemia (1600–2200 mg/dL of plasma cholesterol) and atheroma lesions in the vascular wall [53]. Although it was initially thought that LDLr-/- mice needed cholate to develop atherosclerosis, later studies showed that dietary cholesterol was sufficient to induce atherogenesis [54]. Addition of 0.5% and 1.25% of cholesterol to a high-fat diet (40% kcal from lipid source) resulted in lesion plaque formation in a dose-dependent fashion in LDLr-/- mice. In another study, the effect of dietary cholesterol was evaluated in LDLr-/- mice fed either a Western-type diet, 0.06% cholesterol/21% milk fat, or a cholesterol-enriched diet (1% cholesterol/4.4% fat) for 28 weeks [55]. Atherosclerotic surface areas in the entire aorta were similar in both dietary regimens, but in aortic cross sections the lesions were larger in mice fed the Western-type diet. Surprisingly, lesion morphology was indistinguishable between mice treated either with a Western-type or cholesterol-enriched diet. Therefore, this study led to the conclusion that LDLr-/- mice can develop extensive atherosclerosis when fed a 1% cholesterol diet, without other added fat or cholate components which might add confounding metabolic changes such as IR and metabolic syndrome. Another study showed that feeding LDLr-/- mice a carbohydrate-rich diet with 0.15% cholesterol for 24 weeks produced an accumulation of macrophages in adipose tissue and acceleration of atherosclerosis, thus connecting obesity-mediated inflammation and CVD [56].

In order to better characterize the effect of dietary cholesterol and atherosclerosis, Teupser and colleagues performed a study in LDLr-/- mice with C57BL/6J and FVB/NJ backgrounds [57]. Feeding mice a diet containing 4.3% fat in combination with 0.00% or 0.02% cholesterol for 16 weeks induced hypercholesterolemia and lesions in the aortic root and brachiocephalic artery in LDLr-/- mice with a C57BL/6J background. However, addition of dietary cholesterol of 0.15%, 0.30%, or 0.50% resulted in extensive atherosclerosis in the aortic root, brachiocephalic artery, and whole aorta in LDLr-/- mice with a C57BL/6J background. LDLr-/- mice with a FVB/NJ background remain resistant to the development of atheroma lesions regardless of the cholesterol content in the diet.

In LDLr-/- mice added fat did not worsen atherosclerosis formation despite inducing obesity, hypertriglyceridemia, hyperglycemia, and IR, major risk factors for atherosclerosis. However, lesion size was bigger in mice fed 0.15% cholesterol versus 0.03% [58]. Thus, dietary cholesterol might be determinant in vascular lesion formation in LDLr-/- mice.

LDLr-deficient mice have been also used to investigate a time-course atherosclerotic lesion development. LDLr-/- mice subjected to a diet with 21% high-fat and 0.15% of cholesterol developed mild atherosclerosis in both the aortic root and innominate artery before 3 months. However, advanced lesions were observed at 3 months of feeding in the aortic sinus and in the innominate artery between 6 and 9 months [59].

Interestingly, adding 0.2% of cholesterol using a Western type diet induced accumulation of cholesterol in the liver and aggravated aortic root atherosclerosis in LDLr-/- mice that overexpressed ABCA1 in the liver [60]. The mechanisms of increased cholesterol hepatic content pointed to enhanced HDL-C reverse transport but a not proper removal of cholesterol despite upregulated hepatobiliary secretion. The ABCA1 overexpression accelerated atherosclerosis by a delayed catabolism of apoB-containing lipoproteins (VLDL and LDL). These studies pointed to a key ABCA1 in the modulation of hepatic cholesterol and its impact on atherosclerosis.

Interestingly, genetic inactivation of CD36, a major scavenger receptor in macrophage foam cell formation for which deficiency restrains atherosclerosis, did not confer atheroprotection in LDLr-deficient mice when fed a Western-type diet. However, atherogenesis was significantly diminished when LDLr-/-CD36-/- mice were fed a high-cholesterol diet, thus directly connecting dietary cholesterol and CD36-mediated macrophage foam cells [61]. Increased JNK activation in isolated macrophages, enhanced plasmatic proinflammatory cytokine levels, and reactive oxygen species secretion were among the differences between mice fed a cholesterol-rich diet and Western-type diet. Therefore, dietary cholesterol activates inflammatory pathways in LDLr-deficient mice [61].

Adding dietary cholesterol into a high-fat diet in LDLr-/- also deficient in the scavenger receptor class B type I (SRBI), the main HDL receptor, accelerates cardiac and coronary atherosclerosis complications and thrombosis by an inflammatory mechanism. Thus, treatment of LDLr-/-SRBI-/- mice, with a high-fat diet for 12 weeks induced the lowest proportion of occluded coronary arteries when compared with LDLr-/-SRBI-/- mice fed with cholesterol-containing diets. These diets, which included a high-cholesterol, high-fat high-cholesterol, and a high-fat high-cholesterol cholate diet [62] resulted in different rates of death in LDLr-/-SRBI-/- mice, but mice exhibited similar proportions of occluded coronary arteries. Of note, accumulation of platelets in coronary arteries (a sign of thrombotic events) and myocardial fibrosis were also observed in mice fed a high-cholesterol diet. Other changes included increased circulating Ly6C^hi^ monocytes, signaling a more proinflammatory phenotype induced by the presence of dietary cholesterol [62].

#### 3.2.2. Studies in apoE-Deficient Mice

The most important function of apoE is to allow the specific uptake of apoE-particles which include apoB-containing lipoproteins. Based on the evidence of human kindreds with inherited apoE deficiency who developed xanthomatosis and type III hyperlipoproteinemia with elevated cholesterol, two independent laboratories developed apoE-deficient mice. Unlike the above models, apoE-deficient mice develop spontaneous hypercholesterolemia due to accumulation of VLDL/LDL particles in plasma [79,82]. Atheroma lesions in these mice develop in the aortic root and through the whole aorta in regular chow diet [79,82] and lesion formation accelerates with a Western diet [79]. Mice partially deficient in apoE displayed atheroma lesions when challenged for 12 weeks with an atherogenic diet containing 15.8% fat, 1.25% cholesterol, and 0.5% cholate [63]. This latter study suggested that atherogenic diets containing cholesterol might promote atherosclerosis in genetically predisposed humans due to quantitative limitations of different apoE gene polymorphisms.

By treating apoE-deficient mice with a Western-type diet containing 21% fat, 0.15% cholesterol, and 19.5% casein without sodium cholate, Nakashima and colleagues were able to better characterize the time-line of atherosclerotic events which were highly similar to those observed in humans [64]. The lesions were localized along the aortic tree and had characteristics of more advanced plaques which were characterized by a fibrous cap, presence of smooth muscle cells, and adhesion of monocytes.

Because dietary cholesterol in the apoE-/- mouse model generates lesions that are comparable to those observed in humans, numerous studies have employed diet-induced atherosclerosis in apoE-deficient mice.

In the line of studies in LDLr-deficient mice, hepatic overexpression of ABCA1 increased apoB-lipoprotein and HDL levels in plasma as well as aortic root atherosclerosis in apoE-/- mice fed regular chow diet [65]. In contrast, C57Bl mice overexpressing hepatic ABCA1 and fed a cocoa butter diet with 1.25% cholesterol and 0.5% cholic acid for 15 weeks displayed decreased atherosclerosis compared with control mice. These studies indicate a key role of the ABCA1 gene in hepatic cholesterol homeostasis which is dependent on the genetic background.

ApoE-/- mice are also resistant to developing coronary occlusion and myocardial infarction. A long-term diet cholesterol-enriched by supplementation of 0.15% cholesterol into a high-fat (21% lard) diet for 14 months in apoE-/- mice resulted in plaque rupture in the brachiocephalic artery [66], with small lipid-rich plaque overlying more complex lesions with intraplaque hemorrhage. Of note, this type of lesion generated by long-term feeding of apoE-/- with a cholesterol-enriched diet has been proposed to mimic the human process [67].

ApoE-/- mice have been also used to explore dietary manipulation and to define the detrimental effects of dietary cholesterol. A study comparing different fat-enriched diets showed that adding up to 10% fat from unsaturated sources such as extra virgin olive oil (EVOO) reduced lesion formation, while 1% cholesterol in the diet enhanced lesion size. Moreover, combination in the diet of cholesterol and unsaturated fat from EVOO prevented the beneficial effects of the latter [68].

Recent research has shown that dietary cholesterol in mice might as well modulate inflammatory cells and responses. Feeding apoE-/- mice a cholesterol-rich diet increases the number of total circulating monocytes and induces a proinflammatory Ly6C^hi^ phenotype in a dose-dependent manner [69]. High cholesterol levels in apoE-/- mice have been associated with increased proinflammatory Th1 cell response and T cell receptor signaling [70]. ApoE-/- mice that lack an LXR-β nuclear receptor display enhanced autoantibody and B cell production when challenged with a high-cholesterol diet [71]. Globally, these studies underscore the relevance of the immune system in the effect of cholesterol in the atherosclerosis process by modulating the number and responses of circulating immune cells.

#### 3.2.3. Studies in “Humanized” ApoB-100 Transgenic Mice

To mimic the human lipoprotein profile, studies have been also performed in transgenic mice expressing human apolipoproteins [83] in combination with other genetic manipulations. Transgenic mice overexpressing human apoB100, the main apoB in human LDL, fed a diet containing 16% fat and 1.25% of cholesterol for 18 weeks developed severe hypercholesterolemia and atherosclerosis as compared with regular chow diet-fed non-transgenic mice [72]. In a more recent study, adding 0.2% cholesterol was sufficient to induce vascular damage and atherosclerosis in LDLr-/-TgApoB100 [73]. In the latter study, mice were fed either a standard chow diet, a 0.2% cholesterol diet, a high-fat diet, or a high-fat 0.2% cholesterol diet for 6 months. Comparison of all four diets used in the study indicated that while dietary triglyceride has a modest effect on atherosclerosis addition of dietary cholesterol accelerates lesion formation in LDLr-/-TgApoB100 [73].

#### 3.2.4. Studies in “Humanized” ApoE3*Leiden Transgenic Mice

ApoE*3Leiden transgenic mice, which overexpress a variant of apoE with low affinity for LDLr, develop hypercholesterolemia and atherosclerotic lesions in the whole aorta and carotid arteries when fed a high cholesterol diet in a dose-dependent manner [74]. In these mice, comparing the transcriptomic profiles from mice fed 0%, 0.25%, and 1.25% cholesterol revealed that dietary cholesterol produces hypercholesterolemia and atherosclerosis by inducing a change in the liver from a resilient state into a proinflammatory and proatherosclerotic state [75]. Interestingly, the hepatic resilience induced was attributed to metabolic genes such as SREBP-1/-2, SP-1, RXR, and PPARα. However, the stress induced by high cholesterol levels was associated with pro-inflammatory and pro-atherosclerotic proteins including NFkB, STAT1/3/5, SMAD3, AP-1 (c-jun/c-fos), complement cascade components, chemoattractant factors (ccl6, ccl12, ccl19), chemoattractant receptors (CCR2, CCR5), cytokines (IFN-γ), adhesion regulators, transforming growth factor (TGF)-β, and proteases.

## 4. Conclusions: Impact of Dietary Cholesterol on Inflammatory Processes Associated with NAFLD and ATHEROSCLEROSIS

**Figure 1:**
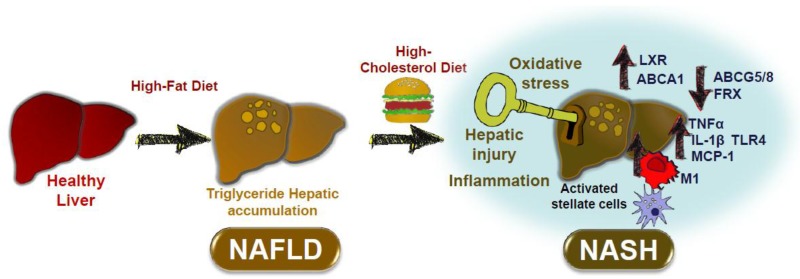
Effect of dietary cholesterol on the progression of NAFLD to NASH. Non-alcoholic fatty liver disease (NAFLD) is characterized by hepatic lipid accumulation. A high-cholesterol diet promotes the progression toward non-alcoholic steatohepatitis (NASH) by several key mechanisms including hepatic stellate cell activation, oxidative stress, activation of inflammatory pathways and hepatocyte death. Dietary cholesterol promotes NASH by modulating the expression of hepatic metabolic genes such as *FXR* and *ABCG5/8*, as well as the expression of cytokines like TNFα, IL-1β, and MCP1. A change in macrophage phenotype in resident macrophages or Kupffer cells toward a M1 phenotype has been suggested. Other proposed mechanisms include the activation of TLR4-dependent pathways and the upregulation of ABCA1 by LXR nuclear receptors.

In animal studies dietary cholesterol might be a key factor in the progression from NAFLD to NASH. The main mechanisms related to this are shown in Figure 1. Hepatic triglyceride accumulation is asymptomatic, but under certain conditions dietary cholesterol might promote the transition from NAFLD to NASH. Dietary cholesterol might activate hepatic injury by increasing the M1 phenotype in Kupffer cells, with activation of hepatic stellate cells, oxidative stress, and inflammation (TNFα, MCP1, IL-1β, and TLR4). Deranged regulation of hepatic genes, such as ABCA1, ABCG5/8, and LXR by dietary cholesterol might also promote further hepatic cholesterol accumulation and hepatic stress.

**Figure 2:**
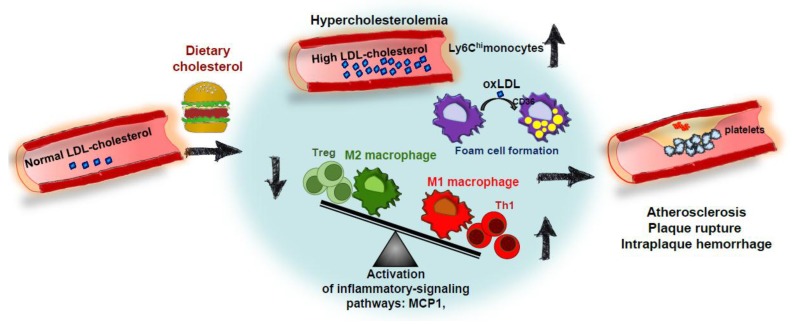
Impact of dietary cholesterol on atherosclerosis. High circulating cholesterol levels (mostly cholesterol carried by LDL-C particles) are the main risk factor for developing atherosclerosis. Under certain conditions, dietary cholesterol induces hypercholesterolemia and enhanced levels of proinflammatory Ly6C^hi^ monocytes. This might facilitate macrophage foam cell formation through the uptake of modified LDL particles by scavenger receptors (CD36). Atherosclerosis development involves an imbalance between M1 macrophages/M2 macrophages and Th1/Treg cells which can be facilitated by hypercholesterolemia. These events lead to the activation of inflammatory pathways and the progression of disease toward more advanced plaques and, in some cases, plaque rupture, intraplaque hemorrhage, and thrombosis. LDL-C: low-density lipoprotein cholesterol

Hypercholesterolemia (especially high LDL-C) promotes CVD progression. However, the impact of dietary cholesterol on this disease is highly dependent on the ability to dietary cholesterol to modulate plasmatic cholesterol levels [84]. Although dietary cholesterol studies in animal models may not directly translate to humans, they have been useful to better understand the mechanisms of the process. Thus, as pointed out above, manipulation of dietary components has been employed to induce atherosclerosis in LDLr-/- mice, to speed up atheroma plaque formation animal models, to “humanize” lipoprotein profiles, to stress hepatic cholesterol metabolism for wide metabolomic screenings, and to induce coronary artery atherosclerosis and vulnerable plaques. Figure 2 displays the main determinants by which cholesterol in the diet accelerates atheroma plaque formations. Dietary cholesterol produces hypercholesterolemia and may increase circulating levels of pro-inflammatory Ly6C^hi^ monocytes. This environment facilitates foam cell formation by uptake of modified LDL particles by CD36 scavenger receptors, among others. Deranged balance between pro-inflammatory Th1 and Treg cells and polarization of macrophages toward a pro-inflammatory M1 phenotype also lead to secretion of inflammatory mediators inducing the formation of advanced plaques, which are vulnerable to rupture, hemorrhage and thrombus formation.

In some experimental settings the relevance of hypercholesterolemia might be overruled. Studies in genetically-modified mice, like those with tissue-specific deficiencies or gain-of-function, showed that high cholesterol diets might not be sufficient to induce atherosclerosis. This is the case for mice with genetic manipulation in relevant inflammatory genes and cells, which modulate atherosclerosis without changing plasmatic cholesterol levels. For example, deficiency in any of the components of the MCP1/CCR2 proinflammatory axis (a main inflammatory pathway in the recruitment of monocyte-derived macrophages in atheroma lesions) in LDLr-/- or apoE-/- or in transgenic human apoB mouse models decreases atherosclerosis development despite high-cholesterol feeding [85]. It is well-known that cholesterol accumulation in the subendothelial space and in plasma becomes toxic to immune cells by promoting inflammatory responses that might become chronic [86] and induce cell death processes [87]. In fact, in the last decade the discovery of novel inflammatory pathways has provided an understanding of the connection between cholesterol metabolism and immune cells [88]; however, discussion of this research is beyond the scope of this review. All this new information points to immune cell modulation as a primary target for many therapeutic strategies that are currently under development in preclinical and clinical studies [9,89,90].

In summary, studies in animal models suggest that in order to gain benefit from dietary cholesterol restriction several factors should be considered. One limitation of these studies to bear in mind is that the cholesterol dietary content used in animal studies is about 60-fold the cholesterol daily consumed by humans. Therefore, a main conclusion that can be drawn from these studies is the use of moderate cholesterol-content diets for future experimental studies in metabolic chronic diseases. Moreover, caution must be taken with the mechanisms described in these studies as they might not directly be translated into human pathology. For NAFLD, dietary cholesterol might directly induce inflammatory stress in liver and promote hepatic dysfunction, therefore, under certain conditions cholesterol restriction would be beneficial. On the contrary, for atherosclerosis and CVD, the benefits of dietary cholesterol restriction largely depend on hepatic metabolic regulation and on how this cholesterol translates into plasmatic cholesterol levels. Despite the use of lipid-lowering strategies, CVD acute events are observed in patients at risk, meaning that although cholesterol levels have to be kept as low as possible, other therapeutic strategies are needed to restrain the progression of this metabolic disease.

## Figures and Tables

**Table 1:** Impact of dietary cholesterol in rat models of non-alcoholic fatty liver disease (NAFLD) and non-alcoholic steatohepatitis (NASH).

Study/Animal Model	Diet	Age	Stage Disease	Mechanism
Cote el al., 2013 [19] Sprague–Dawley rats	40% fat and 1.25% cholesterol	8-week-old female	Fatty liver disease	Hepatic accumulation triglycerides and cholesterol Decreased FXRs Lower expression of HMG-CoA-r, FDFT1, and ABCG8
Ichimura et al., 2015 [20] Sprague–Dawley rats	Fat alone or in combination with 1.25% or 2.5% cholesterol	9-week-old male	Hepatic steatosis	Diminished CPT activity and ABCG5
Moriya et al., 2012 [21] SHRSP5/Dmcr rats	High-fat diet	10-week-old male	Hepatic fibrotic and inflammatory status of NASH	Altered TNFα proinflammatory cytokine and NFkB pathways
Yeti et al., 2013 [22] SHRSP5/Dmcr rats	Fat and cholesterol High-fat high-cholesterol diet	Male SHRSP5/Dmcr rats at 10 weeks old	Phenotype similar to NASH in humans Inflammatory fibrotic liver disease Hepatocyte necrosis	Downregulation of caspase activity
Horai et al., 2016 [23] SHRSP5/Dmcr rats	High cholesterol	6-week-old male rats	Hepatic steatosis, inflammation, and fibrosis	Eosinophilic inclusion bodies and mega-mitochondria

HMG-CoA-r: 3-hydroxy-3-methylglutaryl coenzyme A reductase; FXR: farnesoid X receptor; FDFT1: farnesyldiphosphate farnesyl-transferase 1; ABCG8: adenosine triphosphate-binding cassette transporter G8; NFkB: nuclear factor kappa B; CPT: carnitine palmitoyltransferase.

**Table 2:** Impact of dietary cholesterol in mouse models of non-alcoholic fatty liver disease (NAFLD) and non-alcoholic steatohepatitis.

Study/Mouse Model	Diet	Age	Stage Disease	Mechanism
C57BL/6J				
Matsuzawa et al., 2007 [8] C57BL/6J mice	1.25% cholesterol and two different amounts fat (7.5% and 60%)	Males at 6 weeks of age	Insulin resistance Fibrosis Steatohepatitis	Down-regulation of antioxidant enzymes
Savard et al., 2013 [24] C57BL/6J	15% fat or 1% cholesterol	30 weeks	Hepatic fat accumulation	
Neuschwander-Tetri et al., 2013 [25] C57BL/6J mice	15% fat and 1% cholesterol	30 weeks	Severe steatosis Inflammation Fibrosis	Inappropriate suppression of fatty acid β-oxidation
Vergnes et al., 2003 [26] C57BL/6J	Fat, cholate, and/or cholesterol	At 3 months of age, mice were fed with the specified diet for 3 weeks	Hepatic steatohepatitis	Activation of hepatic stellate cells, SAA family genes, histocompatibility antigens, Il2rγ, Scyb9, Samhd1
Desai et al., 2008 [27] C57BL/6J	1.25% cholesterol, 0.5% cholic acid, and 16% fat	Males at 8–10 weeks of age were fed for 3 weeks with the diet	Hepatic steatohepatitis	Mononuclear leukocyte infiltration in liver Enhanced MCP1, RANTES, MIP2
Sumiyoshi et al., 2010 [28] C57BL/6 mice	15% milk fat, 1.5% cholesterol and 0.1% cholic acid	Males at 4 weeks old were fed with diet for 25 or 55 weeks	Hepatic steatosis Fibrosis Tumor formation (focal nodular hyperplasia)	Elevated levels of MCP1 levels and PDGF-B protein
Ganz et al., 2015 [29] C57BL/6	High fat, high cholesterol and high sugar supplement	Males aged 8–10 weeks old were fed with diet for 8, 27, or 49 weeks	Hepatic steatosis at early stage Fibrosis and characteristics of NASH at a late stage	Enhanced levels of MCP1, TNFα, and IL-1β Macrophage polarization toward an M1
Tu et al., 2017 [30] C57BL/6J	High-fat high-cholesterol and cholate diet	Males and females at 8 weeks of age fed their respective diets for 3 weeks	Hepatic pathology similar to NAFLD and NASH	Elevated free cholesterol, cholesterol esters, and cholic acid Changes to metabolism of sphingomyelins and phosphatidylcholines
Studies in lipase-deficient mice				
ATGL-/- [31,32,33,34]	High-fat high-cholesterol diet	2–12 months old	Severe hepatic steatosis	Activation of inflammatory pathways
Andres-Blasco et al., 2015 [35] HL-/-	High-fat high-cholesterol diet	At two months of age, mice were fed for 16 weeks with diet	Hepatic steatosis and liver inflammation	Dyslipidemia Increased NEFA Enhanced macrophages Circulating levels of MCP1 and Th17 T-cell subset
Chiu et al., HL-/-, 2010 [36]	High-fat 21% diet and 0.15% cholesterol	Females 21–23 weeks old	Decreased hepatic steatosis	No dyslipidemia and IR
Studies in low-density lipoprotein receptor and apolipoprotein E-deficient mice				
Wouters et al., 2008 [3] LDLr-deficient and apoE2 knock-in	High-fat diet with cholesterol	Males or/and females were fed for 2, 4, 7, and 21 days or for 7 days according to experiments	NASH Hepatic steatosis with inflammation	
Subramanian et al., 2011 [37] LDLr-deficient	Fat, carbohydrate and cholesterol	10-week-old males were fed for 24 weeks with diet	NASH	Macrovesicular steatosis, inflammatory cell foci and fibrosis
Prieur et al., 2010 [38] LDLr-deficient mice	Diet enriched in fat, carbohydrate and cholesterol	Males at 10 weeks of age were fed for 24 weeks with diet	Hepatic inflammation	Hepatic macrophage infiltration, apoptosis, and oxidative stress.
Van Rooyen et al., 2011 [39]. Alms1 mutant (foz/foz) and wild-type diabetes NOD.B10,	Dietary cholesterol	Females at 8 weeks of age were fed for 12 or 24 weeks with diet	Hepatic free cholesterol accumulation	Increased macrophage, liver apoptosis and fibrosis
Schierwagen et al., 2015 [40]. apoE-/-	Western-type diet containing 1.25% of cholesterol	12 weeks age + 7 weeks diet	Phenotype resembling that of human NASH.	Hepatic fibrosis Upregulation of TGFβ Increased hepatic collagen Activation of hepatic stellate cells
Rodríguez Sanabria et al., 2010 [41] apoE-/- vs LDLr-/-	20% fat and 0.25% cholesterol	Males 10 weeks age + 6 weeks diet	Inflammation vs. fatty liver	Increased macrophage and inflammatory nodules (apolipoprotein E, apoE-/-) vs. hepatic steatosis (LDLr-/-)
Kampschulte et al., 2014 [42] apoE-/-LDLr-/-	Western diet containing 5% cholesterol and 21% or regular chow control diet	Males at 4 weeks of age were fed for 35 weeks with diet	Hepatic steatosis Fibrosis Hepatocellular injury	Macrophage and T cell infiltration, hepatic ROS accumulation, JNK activation Induction of PPAR-*α*

TGF: transforming growth factor; Il2rγ: interleukin-2 receptor γ; MCP1: monocyte chemotactic protein 1; RANTES: regulated on activation normal T cell expressed and secreted; MIP2: macrophage inflammatory protein 2; SAA: serum amyloid A; Scyb9: small inducible cytokine B9; Samhd1: SAM domain and HD domain 1; PDGF-B: platelet-derived growth factor B; ROS: reactive oxygen species; JNK: c-Jun N-terminal kinase; PPAR-*α*: peroxisome proliferator-activated receptor *α*; LDLr: low-density lipoprotein receptor.

**Table 3:** Impact of dietary cholesterol in mouse models of atherosclerosis.

Study/Mouse Model	Diet	Age	Stage of Disease
C57BL/6J			
Lee et al., 2017 [51]	30% fat, 5% cholesterol and 2% cholic acid		Increased serum cholesterol levels Atheroma lesions and extreme hepatic damage
Paigen et al., 1987 [52] C57BL/6 mouse	1.25% cholesterol, 15% fat, and 0.5% cholic acid (toxic atherogenic diet)	Diet for 14 weeks	Atheroma lesions Discrete hypercholesterolemia
Vergnes et al., 2003 [26]. C57BL mice	Different diets (1.25% cholesterol, 0.5% sodium cholate, and 7.5% cocoa butter, and three other diets which omitted one of the three components of the atherogenic diet)	Males at 3 months of age were fed with the diet for 3 weeks	Dietary cholesterol induces the expression of inflammatory genes Cholate induces the expression of extracellular matrix deposition genes such as collagen
Studies in LDLr-deficient mice			
Ishibashi et al., 1994 [53].	1.25% cholesterol, 7.5% cocoa butter, 7.5% casein, and 0.5% cholic acid	Diet for 6, 7, or 8 months	Severe hypercholesterolemia Atheroma lesions in the vascular wall
Lichtman et al., 1999 [54]	0.5% and 1.25% of cholesterol to a high-fat diet	Males at 8 to 12 weeks of age were fed with the diet for 12 weeks	Lesion plaque formation in a dose-dependent manner
Hartvigsen et al., 2007 [55]	Western-type diet, 0.06% cholesterol/21% milk fat, or a cholesterol-enriched diet, 1% cholesterol/4.4% fat	Males were fed with the diet for 28 weeks	Atherosclerotic lesions
Subramanian et al., 2008 [56]	Carbohydrate-rich diet with 0.15% cholesterol	Males at 8 weeks old were fed with diet for 24 weeks	Accumulation of macrophages in adipose tissue Acceleration of atherosclerosis
Teupser et al. 2003 [57] LDLr-/- mice in C57BL/6J	4.3% fat in combination with 0.02% 0.15%, 0.30%, or 0.50% cholesterol	Mice at 28 days of age were fed the diet for 16 weeks (20 weeks of age)	Atherosclerosis in aortic root, brachiocephalic artery and whole aorta
Wu et al., 2006 [58]	0.15% cholesterol versus 0.03% cholesterol with high fat in each diet	Diet 20 or 40 weeks	Lesion size was bigger in mice fed 0.15% cholesterol versus 0.03% cholesterol Addition of fat to a cholesterol-rich diet did not increase atherosclerotic lesion hypertriglyceridemia
Ma et al., 2012 [59]	21% fat, 0.15% cholesterol	Males at 8 weeks were fed with the diet for 1, 3, 6, 9, 12 months	Before 3 months: slight atherosclerotic lesions in aortic roots and innominate artery At 3 months: advanced lesions in the aortic sinus At 6–9 months: advanced lesions in the innominate artery
Joyce et al., 2006 [60]	0.02% cholesterol and 4% fat or 0.2% cholesterol and 21.2% fat	Diet 4, 9, or 12 weeks prior to sacrifice	Increased hepatic content of cholesterol and aggravated aortic root atherosclerosis in LDLr-/- mice that overexpressed ABCA1 in the liver
Kennedy et al., 2009 [61] LDLr-/-CD36-/- mice	High-cholesterol diet	Diet for 12 weeks	Atherosclerotic lesions
Fuller et al., 2014 [62]. LDLr-/- SRBI-/-	High-cholesterol high-fat high-cholesterol high-fat high-cholesterol cholate diet	Females at 10–12 weeks of age were fed 12 weeks with the atherogenic diet	Occluded coronary arteries Accumulation of platelets in atherosclerotic plaque in coronary arteries (thrombosis) Myocardial fibrosis Increased circulating Ly6C^hi^ monocytes
Studies in apoE-deficient mice			
Zhang et al., 1994 [63] Partially deficient in apoE	15.8% fat, 1.25% cholesterol, and 0.5% cholate	Beginning at 8 weeks of age, mice were fed 6 or 12 weeks with the diet	Atheroma lesions
Nakashima et al., 1994 [64]	Western-type diet, containing 21% fat, 0.15% cholesterol and 19.5% casein without sodium cholate	Males at 5 weeks of age were fed the diet until 6, 8, 10, 15, 20, 30, and 40 weeks of age	Advanced plaques Development of fibrous cap, presence of smooth muscle cell and adhesion of monocytes
Joyce et al., 2002 [65] ABCA1-Tg apoE-/-	1.25% cholesterol and 0.5% cholic acid	Mice at 2–3 months of age were maintained on the diet for 15 weeks before sacrifice	Overexpression of ABCA1 increased apoB-lipoprotein and HDL levels in plasma and reduced atherosclerosis in vivo
Johnson et al., 2001 [66]	0.15% cholesterol into a high-fat diet	Seven-week-old mice were fed the diet for 14 months	Plaque rupture in the brachiocephalic artery Lesions with intraplaque hemorrhage
Bond et al., 2011 [67].	Cholesterol diet		Lesions in the brachiocephalic artery
Acin et al., 2005 [68]	Different fat-enriched diet and cholesterol with or no unsaturated sources such as extra virgin olive oil (EVOO)	2-month-old mice were fed with different diets for 10 weeks	Reduced lesion formation with EVOO Cholesterol prevented the beneficial effects of unsaturated fat from EVOO
Swirski et al., 2007 [69]	Cholesterol diet	Beginning at 10 weeks of age, mice were fed 20–25 weeks with the diet	Accelerated atherosclerosis by increasing Ly6C^hi^
Mailer et al., 2017 [70]	Cholesterol diet	Mice at 8–10 weeks of age were fed for 4, 12, or 24 weeks with the diet	Accelerated atherosclerosis by activating T cell receptor signaling
Ito et al., 2016 (in combination with LXRβ-/-) [71]	Cholesterol diet	Diet for 8, 12, or 16 weeks	Accelerated atherosclerosis and production of autoantibodies and B cell expansion
Studies in “humanized” ApoB-100 and ApoE3*Leiden transgenic mice			
Purcell-Huynh et al., 1998 [72]	16% fat and 1.25% cholesterol	Males and females at 5 weeks of age were fed the diet for 5 or 8 weeks	Severe hypercholesterolemia and atherosclerosis
Laplante et al. 2013 [73] LDLr-/-TgApoB100 and LDLr-/TgApoB100IGFII	Standard chow, 0.2% cholesterol diet, high-fat diet or high-fat 0.2% cholesterol diet	Males aged 6 weeks were fed the diet for 6 months	Cholesterol accelerates lesion formation in both LDLr-/-TgApoB100 and LDLr-/-TgApoB100IGFII.
Van Vlijmen et al., 1994 [74] ApoE*3Leiden transgenic mice	High cholesterol	At 8–10 weeks old age, mice were fed for 6 weeks with the diet	Hypercholesterolemia and atherosclerotic lesions in the whole aorta and carotid arteries
Kleemann et al., 2007 [75] ApoE*3Leiden transgenic mice	0%, 0.25%, and 1.25% cholesterol	Female E3L mice at 12 weeks old were treated with diet for 10 weeks	Hypercholesterolemia and atherosclerosis

## Abbreviations

ABCG8:	adenosine triphosphate-binding cassette transporter G8
ABCG5:	adenosine triphosphate-binding cassette transporter G5
ApoE:	apolipoprotein E
ATGL:	adipose triglyceride lipase
CPT:	carnitine palmitoyltransferase,
FDFT1:	farnesyldiphosphate farnesyl-transferase 1
FXR:	farnesoid X receptor
HMG-CoA-r:	3-hydroxy-3-methylglutaryl coenzyme A reductase
Il2rγ:	interleukin-2 receptor γ
LDL:	low-density lipoprotein
LDL-C:	low-density lipoprotein cholesterol
LDLr:	low-density lipoprotein receptor
LXR:	liver X receptor
MCP1:	monocyte chemotactic protein 1
MIP2:	macrophage inflammatory protein 2
MTTP:	microsomal triglyceride transfer protein
NFkB:	nuclear factor kappa B
PDGF-B:	platelet-derived growth factor protein B
PPAR-α:	peroxisome proliferator-activated receptor
RANTES:	regulated on activation normal T cell expressed and secreted
ROS:	reactive oxygen species
SAA:	serum amyloid A
